# Sustainable medical research by effective and comprehensive medical skills: overcoming the frontiers by predictive, preventive and personalized medicine

**DOI:** 10.1186/1878-5085-5-14

**Published:** 2014-08-27

**Authors:** Guglielmo M Trovato

**Affiliations:** 1 Dipartimento di Scienze Mediche e Pediatriche, Unità di Terapia e Diagnostica Medica non Invasiva AOU Policlinico-VE, University of Catania, 95124 Catania, Italy

**Keywords:** Sustainable medical research, Medical skills, PPPM

## Abstract

**Background:**

Clinical research and practice require affordable objectives, sustainable tools, rewarding training strategies and meaningful collaboration.

**Method:**

Our unit delivers courses on project design and management promoting ideas, useful skills, teaching and exploring implementation of networks and existing collaborations. We investigated the effectiveness of a sustainable approach of comprehensive diagnosis and care and its usefulness within concrete models of research project teaching methodology.

**Results:**

The model of predictive, preventive and personalized medicine (PPPM) of adolescent hypertension, developed since 1976 and still active, was displayed. This is a paradigm of comprehensive PPPM aimed at the management of a recognized, but actually neglected, societal and clinical problem. The second model was addressed to the analysis of performance of an outpatient diagnostic and therapy unit and its relationship with the emergency department. Part of the patients, 4,057 cancer patients presenting at the emergency care, were addressed to the outpatient diagnostic and therapy unit for further assessment, treatment and follow-up. The stay in DH was 6.3 ± 2.1 non-consecutive days, with shortage of costs, vs. in-hospital stays. Research planning courses, based on these models, ensued in an increase of competitive project submission and successful funding.

**Discussion:**

Active promotion of interdisciplinary knowledge and skills is warranted. Misleading messages and information are detrimental not only to healthy and sick people but, equally, to all health professionals: efforts for basing on evidence by research any statement are needed. The actual pre-requisite of personalized medicine is the coherent and articulated promotion of the professional quality of staff. Health professionals should and can be skilled in sustainable non-invasive diagnostic procedures, in non-pharmacological intervention, in translational research (from epidemiology to personalized therapy) and in timely dissemination of the information.

**Conclusion:**

Recommendations are provided according to PPPM: proposed models are based on financial sustainability and patient's satisfaction criteria and are addressed to research projects and dissemination also by e-learning. The guidelines of the EU calls in personalized medicine are able to provide a critical added value by accurate planning, transparency of assessment and unbiased reports, dissemination and exploitation.

## Overview

### Beyond the boundaries

**Figure 1:**
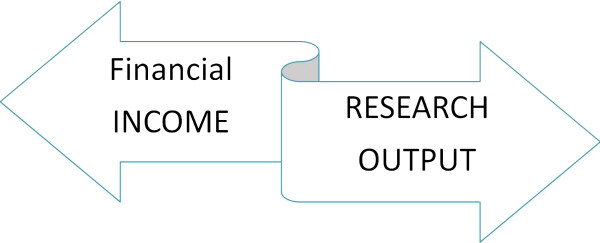
Dual governance situation and divergent drive for research in medicine: a bottom-up need and appeal to research and innovation, stemming by actual expertise and ideas, is concealed, if not counteracted, by the top-down pressure toward researchers for reaching positive financial effects for the individual institutions.

In Italy, and without any great difference in Europe, clinical research is not actively promoted in most institutions: academic-, hospital- and primary care-based organizations seek funding without a realistic knowledge and balance of their available human resources and facilities. Most research is industry- and marketing-driven within the umbrella of clinical trials. The market of research is in part doped by commercial promotions disguised as innovative investigation: the drive of the institutional side is present, often not sufficiently innovative, sometimes overambitious and unrealistic, and very frequently contradictory in its aims, procedures and human resource promotion.This is the result of a dual governance situation (Figure 1). There is a bottom-up need and appeal to research and innovation, stemming by actual expertise and ideas; this is concealed by the top-down pressure toward researchers for reaching favorable effects for the individual institutions. There is the explicit wish of some financial income, which will be achieved along with some benefit in the quality of public perception of quality and innovation of that particular institution or activity.

**Figure 2:**
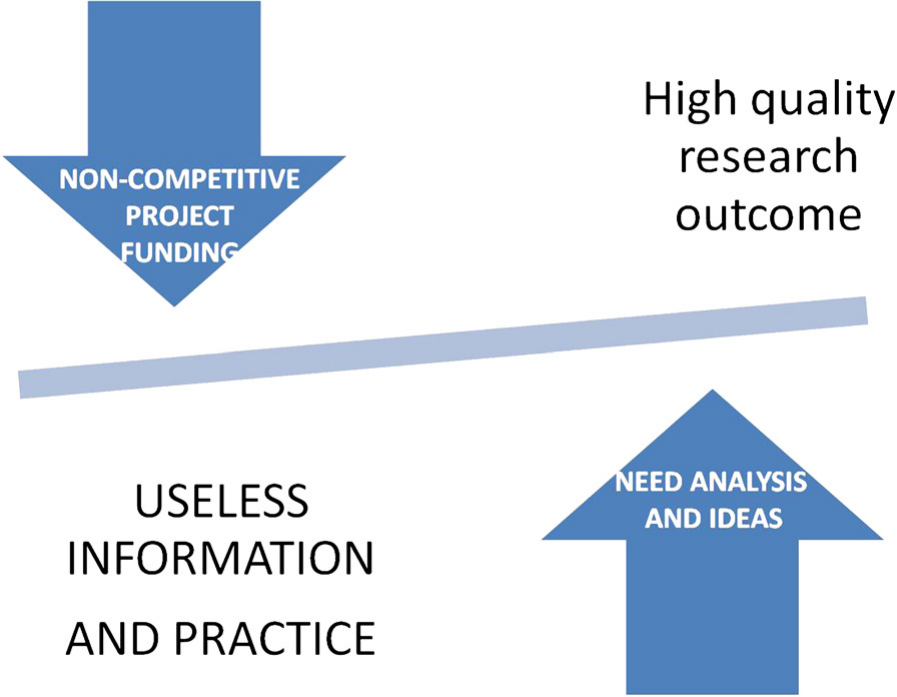
The balance of quality and financial benefits for the hosting institution depends critically on the type of application: non-competitive calls, which are often a privilege of research units and institutions with a greater political support, are likely to deliver less innovative projects and outcomes; competitive call, appealing also to ideas, are more suitable to be able to deliver groundbreaking results and research.

Nonetheless, the actual commitment of the health organization managers is often limited and, if any, is overwhelmed by the concern that human resources and facilities could be diverted toward useless intellectual exercises; there is the concern that, by this work, health professionals will waste or, at least, lose time subtracting strengths that should be addressed to the daily work of health care or practical training. Moreover, despite the great efforts of training people in research planning, research managing and research fundraising, only few more skilled institutions are actually frequent applicants with a relevant yield of successful proposal. Project making is an expert work and the key factors for a successful application getting funding are many, but, among them, the previous concrete experience of most or all the researchers in applying for grants: this expertise was often achieved abroad, not only in Europe but, more frequently, in the USA.National funding, even always with an increasingly shortage, is often only partially competitive: this is a misguiding practice and lesson for most researchers. Probably, this is a consequence of our mistrust of the future which makes it hard to give up the past. The road for going beyond the current practice, notwithstanding the lack of effectiveness or of best evidence of process and outcome, is difficult to pursue without a culture that favors some type of skilled human resource mining. Nonetheless, without any obvious rationale, there is a great appeal to fundraising efforts and strategy, despite that this would require an open and respectful approach to independent thinking and not only efforts for getting money without a pragmatic innovation strategy (Figure 2).

Actually, funding is the pre-requisite for any research, and when no specific support is available, this could become a barrier to the development of articulated independent research, from epidemiology to personalized therapy. It is our current experience that research which is not funded under competitive and transparent procedure is likely to be of minimal impact, often redundant, time-wasting and substantially useless. It is sufficiently accepted that science is not an illusion. But an illusion it would be to suppose that what science cannot give us we can get elsewhere and otherwise.

Despite the need of a close managed drive of any research project, also with the added barriers of ethical committee, most good-quality clinical researches, which are published on the best rated peer-reviewed journals, are not solicited and not specifically supported by non-profit institutions and, often, by industries or private enterprises.

Differently, as a consequence of the need of publishing or perishing, most fund-supported clinical researches address their reports to open-access quickly publishing journals, instead of the more reputed and high-impact ranked journals. This is the case of the most ‘scientific’ exercises, since many of the national and local funding resources are still devoted to the so-called ‘innovation’ , too often only incomplete application of practice and tools that are already in use elsewhere. This type of research is not suitable to be adequately disseminated outside the local level since it is, seemingly, too often not appropriate for submission to the peer-reviewed filters of independent editorial offices. Academic research has too often the features of a tool devised more for enhancing niche knowledge and career progress than as an actual resource for promoting quality in health care and advancement in knowledge.

This line of ignoring the possible advantages of clinical research even inside a single institution favors the promotion of the professional quality based increasingly more on compulsory lifelong learning activity, quite theoretical and not sufficiently practical, than on actual training by research, as is more rewarding and, significantly, as required by most criteria of European research calls for funding.

**Figure 3:**
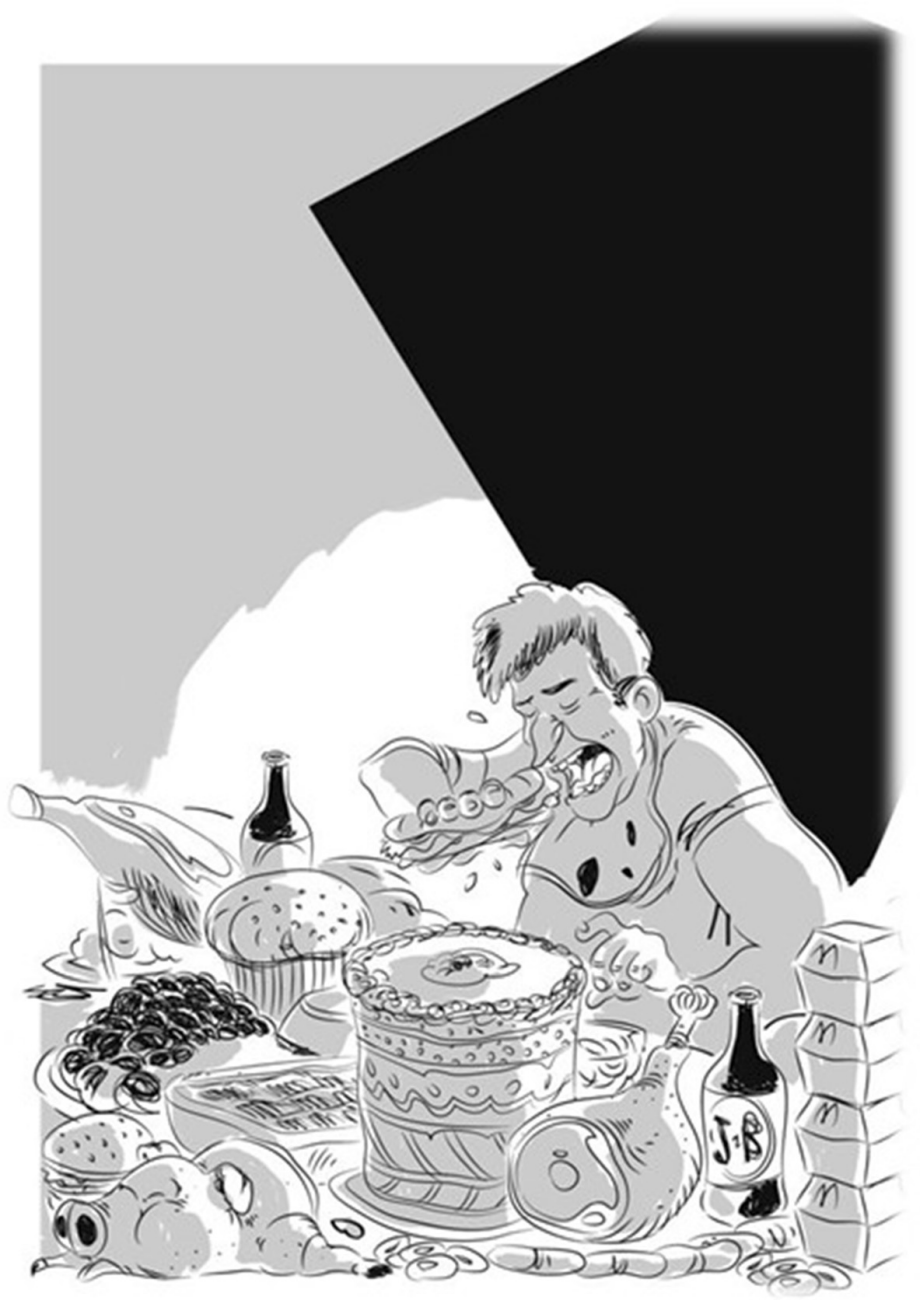
**It is known that unhealthy lifestyles—nutrition, sedentary life, work-sleep shifting, alcohol and tobacco habits, environmental pollution, including noise and light pollution—are among the most critical unhealthy conditions.** Lifestyle intervention, sustainable procedures and therapies are still neglected tools, despite that even the national recommendation and guidelines confirm that these affordable strategies should be developed for enhancing evidence-based medicine and more valuable cost-benefit balance. Environment and behavior are very strictly linked to each other and have not a great sense to address only personal behaviors not considering environment, including the places of daily life and work.

The lack or shortage of actual professionalism in clinical training is well displayed by the ‘teaching pulsion’, which leads to an overstatement of actual skills and knowledge of teachers and instructors. The most evident feature of many training programs is the enthusiasm of new entrant trainers, instructors and supervisors, which by this opportunity skip their daily work, if any, and do not display adequate knowledge, professional skills and scientific background, so that the deliverables are not convincingly merged into comprehensive and effective training strategies. Benefits for trainers are prominent and learners' achievements are, seemingly, conjectural or questionable. It is a frequent ascertainment that too many courses are dealing with the most recent guidelines on best practice, not always sufficiently up-to-date or appropriate, more than on the implementation of innovation through novel available information. Knowledge and practice of e-learning as an effective and powerful tool for teaching, health education and literacy are still far from adequate application, and e-learning is often claimed to be used without an actual practice, expertise and strategy.Lifestyle intervention, healthy nutrition and habits, and promotion of physical exercise against sedentary life are sustainable procedures and therapies. Nonetheless, all are still neglected tools, despite that even the national recommendation and guidelines confirm that these affordable strategies should be developed for enhancing evidence-based medicine and more valuable cost-benefit balance (Figure 3).

In this short overview, the mission and the methodology of clinical research and practice is presented within a synthetic frame, providing concrete examples of affordable clinical researches, of rewarding training strategies and of meaningful research collaboration. It will we developed within the concept of participatory medicine and research (patients, association, professionals outside the health workers' circle), since it is necessary to try to go beyond the boundaries of established practices and beyond the limitations and obstacles that can supervene. The inadequate and/or inappropriate commitments of stakeholders are probably the beginning and the end of many research projects.

### Overcoming the frontiers: a single-institution experience

In the last decade, we developed three yearly postgraduate curricula to promote original thought in clinical research, to provide basic and operationally useful skills, to teach and to explore the possibilities of setting up networks and existing collaborations [1]. These curricula were aligned along the themes of healthy lifestyle and nutrition, e-learning tools and ICT (telemedicine) in health sciences and in clinical diagnostic and intervention ultrasound. All these aspects were included as models within a comprehensive course addressed to the methodology of research project design, planning and implementation. Vocational training was enhanced integrating and boosting the participation of all interested clinical professionals (medical doctors, nurses, technicians, biologists, dietitians, physiotherapists, others) in order to facilitate those who wish to plan projects, intersectoral collaboration and multidisciplinary features. The interaction and contribution of participants are the core of the training methodology, and research is developed as a permanent mode of professional progress. The outcome is measured on the basis of the number of individual competitive projects submitted by trainees for funding to national or international institutions. Results achieved are satisfactory: there was a significant increase (50%) of the number of the projects successfully submitted, which were proposed by 30% of the participants in 2012.

**Figure 4:**
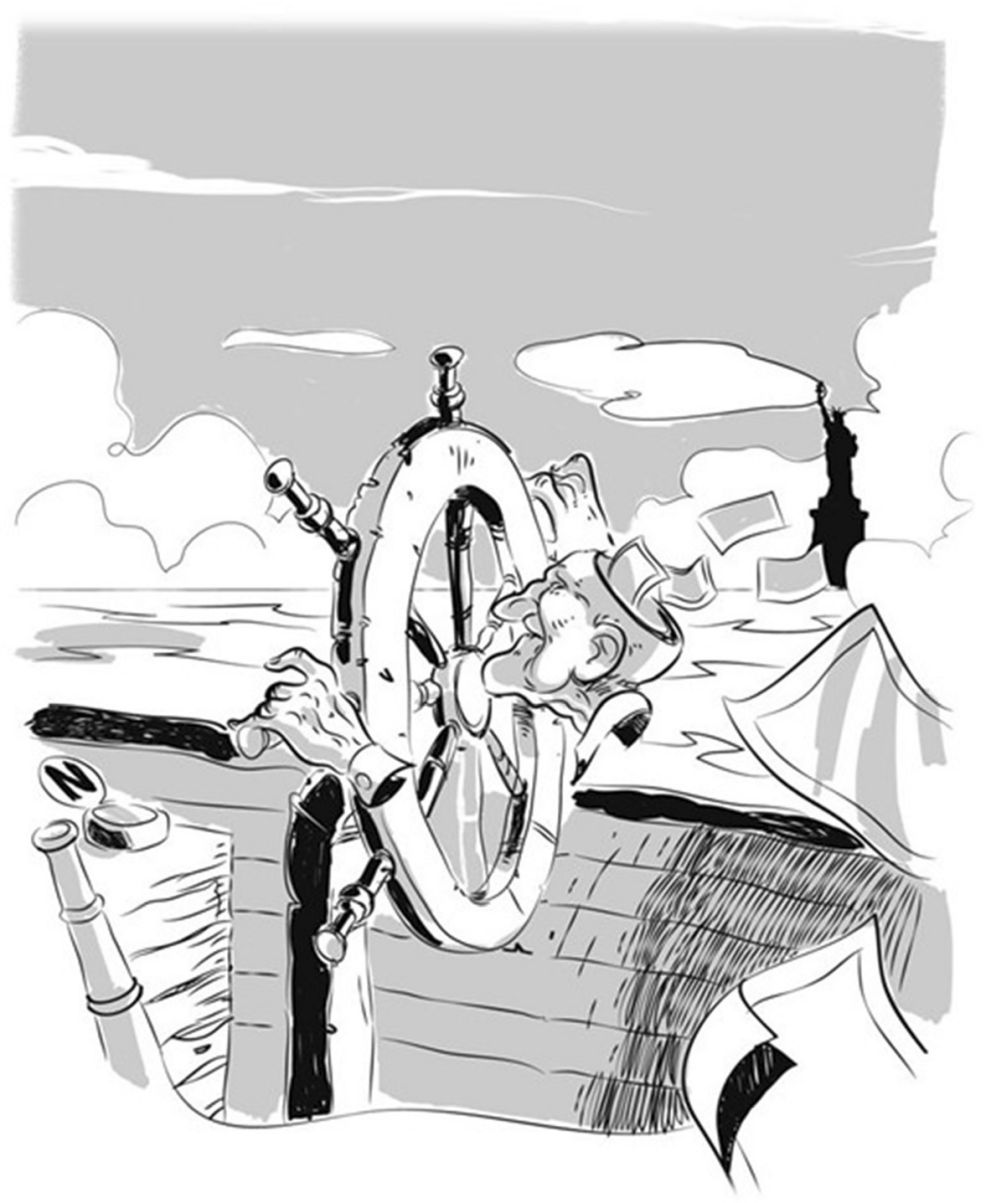
**Overcoming the frontiers, beyond the barriers of established relationships, knowledge and practice, is the aim of any research and innovation.** Exploring by research new territories of knowledge implies some level of forgetfulness of the current practice and concepts. Research is always a risky process, based on actual skills and expertise, but trying to overcome an ocean of misunderstanding and wrong way of navigation. A free mind and ideas are the basis for any research, even a modest one, provided that the support of a clear method and explicit objectives are present.

This preliminary work was done and reached the wished information results:

1. The actual output of researches of the single and joint research groups, published on peer-reviewed journals, was analyzed, identifying as more suitable to further projects those researchers that were able to produce reports with a recognizable clinical impact and by exclusive or prominent local personal leading coordination.

2. A list of submitted proposals was analyzed, after having detected those with successful outcome and those without success.

3. The European, national, regional and institutional mainstream of research line were analyzed, also in agreement to the opportunities of the forthcoming calls, according to the informal indications available.

4. Themes and requirement of the forthcoming calls were linked with the information of the interest of the single researcher or group, and each of them was individually contacted within a general frame of up-to-date, but mostly addressing the theme and the call which, according to our knowledge, could be the object of a submission.

5. The exercise of working to the draft of the first step of the available two-step proposal was actively promoted, in order to train researchers to be synthetic, concrete and realistic and to have in mind how the first-stage evaluators will proceed. This means that the messages should be very clear and explicit, enlightening novelty and credibility of the proposal and displaying well the current knowledge, the envisaged steps beyond the current state of the art, the realistic impacts and a coordinated and articulated frame of complementary and necessary contributions.The goal of overcoming the frontiers, beyond the barriers of established relationships, knowledge and practice, was the goal of our experience (Figure 4). A very important component, preliminary to the establishment of partnership for research, is to allow last year's students and early medical graduates to be seconded, even for short periods of 3–6 months, abroad. This is already possible on volunteer basis, not only using reciprocal training exchange schemes, such as Erasmus at the European level, but also, more important, by the new ‘Erasmus for All’ which, when addressed to non-European countries and, in particular, to Russia, China, USA and New Zealand-Australia, have not the limitation of reciprocity. The advantage of opening to these continents is obvious, since it is more realistic that young medical doctors can appropriately integrate their knowledge and training being exposed to different health and societal systems, transferring in both sense needs and experience potentially fruitful for new ideas and innovation. The strategy for enhancing the excellence in clinical research implies that also at the level of medical students and young graduates the commitment of hosting institution for delivering a robust training in methodology and clinical research should be requested. This is the uniform requirement, participation to predictive, preventive and personalized medicine (PPPM) research, for the seconding of our medical residents still in training when going elsewhere, in our country or abroad.

### Background and objectives

Alternatives to traditional hospital acute care for medical disorders are actively searched since they address benefits for the health systems, which experience budget constraints [2]. The comprehensive approach to the use of high-tech medicine for improved health care provided by the European Association for Predictive, Preventive, and Personalised Medicine (EPMA) is exactly along this line of intervention, aimed to introduce integrative approaches for advanced diagnostics, targeted prevention, treatments tailored to the person and cost-effective health care [3]. The rubber hits the road in the doctor-patient relationship. The collective health care knowledge must be applied to the particular patient's health care situation and is related preliminarily with the design of clinical studies, e.g. for evaluating the clinical utility and robustness of prognostic and predictive biomarkers [4]. The problems in the quality and management of medical services challenging health care systems worldwide depend on the pandemic scenario in the progression of common chronic diseases, delayed interventional approaches of reactive medicine, poor economy of health care systems, lack of specialized educational programs, problematic ethical aspects of treatments as well as inadequate communication among professional groups and policymakers [5]. Sustainable answers are many, among which is the ‘quick diagnosis units’ [6], which are often reported with a limited clinical description [7]. Also, relationships with non-profit organizations and with patients' associations, namely Cancer Survivors Network, are increasing their articulation with in- and outpatients' care facilities, providing peer support which get benefits and endorsement by the qualified reference institutions. Primary health care use, 2 to 5 years after diagnosis of cancer, especially in younger patients without a chronic disease, has a significant increase of involvement and impact worldwide [8]. Given the expected increase in cancer survivor number and, hopefully, lifespan, an increasing amount of aftercare, also in emergency, is foreseeable and actually seen. With these perspectives, the development of multidisciplinary care standards for cancer survivors could be helpful [8]. In our university hospital, we shifted, beginning in 2004, part of the patients addressed by the emergency department toward our autonomous outpatient Diagnosis and Therapy Medical Unit (DTU). Admittances are primarily oriented toward the care of patients with chronic liver disease, secondary anemia, severe malnutrition (including mental anorexia) or extreme obesity and heart failure; however, at last, overall 25% of patients had an associated diagnosis of cancer; 18% of them were diagnosed by biopsies of various organs within our unit as the first confirmatory diagnosis or as subsequent monitoring control [9]. The close link with the emergency department is devised also for overcoming the gap between the need of a multidisciplinary approach and the actual availability of different specialists for an integrated work and, more, to a meaningful decisional synthesis. This is the road suggested by the European Partnership for Action Against Cancer consensus group, which addresses cancer care to a patient-centered approach, not anymore to a disease-focused approach, providing more attention to psychosocial aspects, quality of life, patients' rights and empowerment and survivorship. Multidisciplinary teams are defined as a practical necessity for optimal coordination among health professionals and clear communication with patients [10]. Considering the sustainability of this strategy, and the actual shortage of different expertise with a disposition to mutual coordination, we devised a more realistic approach relying on and trusting in medical doctors with wider and comprehensive skills useful also for this purpose. The aim was to manage comprehensively most patients enhancing knowledge and skills in diagnostic and interventional ultrasound procedures [9], oxygen therapy and delivery systems and assessment of nutrition and physical exercise lifestyle [11], with consequent medical prescription and follow-up, empowered by specific strategies which were developed previously in cerebrovascular disease [12], fatty liver [13] and other conditions [14,15].

## Methods

### DTU—the unit and the clinical research enhancement by a model

**Figure 5:**
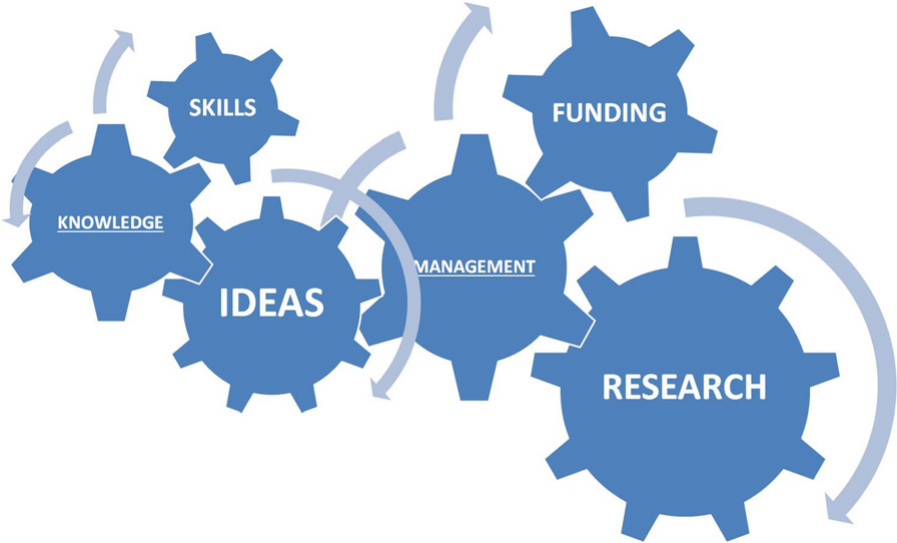
**The research engine.** Relationship and articulation among different components of the research process are displayed.

Our postgraduate teaching and clinical unit, hosted by the university hospital of the School of Medicine, delivers courses on project design and management aimed to promote original thought, to provide basic and operationally useful skills, to teach and to explore the possibilities of setting up networks and existing collaborations (Figure 5). The contribution of ideas, resources, skills and tenacity is the basis for strategic researches aimed at promoting quality, effectiveness and efficiency of health systems. The specific facilities and health care organizations for which the research is a structural mission need quality and visibility, indicators of quality and efficiency. For this reason, training is integrated and participated within all the active components with a clinical profile (doctors, nurses, technicians, biologists, dieticians, physiotherapists, others), in order to facilitate those who wish to plan projects also with international European profile [9]. The interaction and contribution of participants are the core of the training methodology, and research is developed as a permanent mode of professional development. The 3 months 1 day/week (96 h) course on postgraduate medical skills and on research project and methodology, intended as a modality of updating professionalism and expertise in the field of integrated health care organizations, was developed as a modular structure. This course enhances the articulation of medical and surgical areas with the services committed to the evaluation of quality and to preventive and occupational medicine. Moreover, the enrichment of independent thinking for challenging established concepts and ideas addressed also to the reappraisal of already accomplished projects and used as work in progress the available databases of the unit, which are displayed in this report.

Within the course, we are challenging a possible, sustainable and fruitful integration of most of these purposes, e.g. the management of survivor cancer patients referred to the emergency department with a collaboration between outpatient day hospital and emergency facilities (including laboratory and radiology). The model was designed not using a straightforward multidisciplinary approach, i.e. multiple expertise of multiple and integrated units [16], but relying more, if not exclusively, on multi-skilled medical doctors with expertise in clinical medicine, cardiac and general ultrasound (including thoracic ultrasound) and lifestyle assessment and change prescription. This line of approach is also followed, in recent years, by others [17,18]. Of particular interest is that the scientific process around cancer research begins with scientific discovery, followed by development of interventions, and finally delivery of needed interventions to people with cancer. Numerous studies have identified substantial gaps between discovery and delivery in health research. Team science has been identified as a possible solution for closing the discovery to delivery gap [19], identifying the benefits and drawbacks associated with research collaboration along the discovery-development-delivery continuum.

The brief report provided here is the reappraisal of the prevalence of the patients referred to our emergency department with previous, even not definite, cancer diseases, with a focus on the most prevalent types (lung, colonic and breast); it was investigated which of them were subsequently addressed to in-hospital stay, to our comprehensive medical diagnosis unit or to other multidisciplinary outpatient diagnosis.

### A brief report on cancer survivor care: exploring emergency

The outpatient DTU is a comprehensive outpatient autonomous hospital service, which has essential point-of-care laboratory test facility and certified competence of the medical staff (two senior and two junior medical doctors) in medical ultrasound (more than 1,000 ultrasound/year, for each MD, since at least 4 years) and echocardiography. Moreover, dietary/physical exercise assessment and prescription [20,21] are provided by the help of a part-time dietician, a health psychologist and a nurse with intensive care experience. All procedures, including echo-guided diagnostic-therapeutic intervention [22,23], are performed by the components of the staff.

The workup included in all patients a complete ultrasound assessment, including heart, lung, abdomen and thyroid; EKG; focused blood and serological analysis; nutritional (Dietosystem, Milan) and physical activity assessment (Baecke) and subsequent prescription [20]; health psychology strategies [21,24] and pharmacological treatment.

The DTU is a place of training for postgraduate MDs of the School of Internal Medicine and of the Postgraduate School of Medical Ultrasound. The critical contribution of the DTU [9] is medical quality-centered more than medical accountancy-centered. Greater focus on staff professional competences in sustainable diagnostic procedures, such as ultrasound, lifestyle assessment and dietary and health-psychology interventions, is needed for enhancing effectiveness. Such strategy will fasten diagnosis and follow-up, allowing affordable therapeutic personalized approaches. Also, US-guided interventional procedures are an important component of a quicker and more reliable diagnostic and therapeutic process [22,23,25], skipping less reliable procedures and applications [26,27].

Thoracic ultrasound (TUS) image-guided sampling of thickened pleura and of lung nodules is a valuable approach in patients with lung cancer (LC) and malignant pleural mesothelioma (MPM); moreover, we monitor by TUS imaging patients with occupational or environmental risk of MPM and LC, addressing them more selectively to high-resolution computerized tomography (HRCT), also to contrast CT for a further definition [28]. The TUS signs observed in histologically confirmed MPM were irregular thickening of the pleural line (above 5.0 mm) with associated micro-nodules (5–10 mm); a lower percentage showed plaque nodulations (5–10 mm), all with slight or relevant pleural effusion. Patients at risk, without MPM/LG, under TUS monitoring, have irregular thickening of the pleural line (above 3.0 mm), with associated micro-nodules (2–4 mm); a lower percentage has plaque nodulations (4–7 mm). These early signs of lung involvement in subjects exposed to asbestos were observed in the absence of CXR findings and with subsequent HRCT evidence without the need of FNAB. Systemic sclerosis (SSc) is characterized by collagen thickening of the skin and by early and progressive microcirculatory impairment. We investigated and confirmed the concordance between TUS abnormalities, in any phase of disease, and HRCT findings in SSc, concurrently with the association of the nailfold video capillaroscopy (NVC) with TUS and HRCT patterns [29]. Accuracy of TUS at the reliability analysis is high and, with its reproducible measures, is a promising complementary tool for early detection of structural lung abnormalities allowing to refer patients selectively to HRCT or to delay referral. Also, its usefulness and reliability in several emergency conditions is a factor that warrants greatly its more widespread use and more qualified expertise [30,31].

## Results

### Exploring the outpatient clinic added value: the numbers

Throughout the period January 2011 to December 2013, 327,763 patients were referred to the emergency department; of them, 3,303 were cancer patient referrals; considering the absolute number of patients, 2,456 patients were taken in care: 344 for lung, 243 for colonic, 193 for breast, 155 for prostate and 144 for bladder cancer. Moreover, 754 patients were referred for hematological malignant disease; 400 patients, not requiring a specific oncological therapy, were addressed to the outpatient diagnostic and therapy unit for further assessment, treatment and follow-up. The stay in DH was 6.3 ± 2.1 non-consecutive days, with an obvious shortage of costs, in comparison with in-hospital stays.

The promotion of the professional quality in translational research (from epidemiology to personalized therapy) is the pre-requisite for innovative best practices and their dissemination of the most high impact information. As often in research, it is necessary to have a robust background of skills and knowledge, but fostering advancement and innovation can be achieved with an open mind, independent thinking approach. Abandoning ineffective medical practices and mitigating the risks of untested practices are important for improving patient health and containing health care costs [32,33]. The optimal care of cancer survivors, which implies the management of early and late toxicities, should involve consultations with specialists in nutrition, psychiatry, gynecology, pain management, neurology, cardiology and primary care. The access of cancer patients to general emergency can represent a challenge and a conundrum for many physicians and institutions in many situations. Also, the management of emergency by oncologists can be a difficult task, since the hidden effects of cancer can be misinterpreted by excess or by defects. The need of multiple and minor interventions, which are problem-centered and managed by multiple specialists, in comparison with a sustainable and effective patient-centered approach and managed by a comprehensive staff, relies on specialists integrated within a generalist facility and was one of the objects of the course participants' analysis. Models to be proposed aimed to challenge if general health facilities, both general hospital or territorial service, more than inside specialized institutions with a strong focus on oncology, should be delivered not by all-purpose primary care staffs but by effective clinical task forces skilled in the most strategic intervention (non-invasive diagnostic and guided mini-invasive procedures and lifestyle intervention) and which were the available results. The point, and our experience, is that it is expensive to rely on multidisciplinary teams with consultancy referred to oncological care and research units: with this approach, the level of expertise is wider and greater and the feedback fastened with appropriate reliance on daily practice of more generalist facilities. These were the main topics studied and discussed in this regard.

Many of the recommendations outlined, and suitable to be considered ‘deliverables’ , are lifestyle interventions, such as maintaining a healthy body weight, exercise, and routine physician follow-up, which are more easily provided and more easily adhered to within a less specialized context, without the stigma of the cancer patient. Actually, increased risk of cardiovascular disease is a consequence of aging, as it is foreseeable equally in long-term cancer survivors. As a consequence, we are pursuing actively a concrete frame in which follow-up and management are focused not only on both cancer recurrence surveillance and cancer-related complications but also, and even more, on modifying risk factors for comorbid conditions. It is realistic that, by wider dissemination of this practice, outcomes and utilization of health care resources can be optimized with individual surveillance strategies tailored on the knowledge of the most likely natural history of a particular cancer profile, according also to genomic-personalized medicine information [33,34].

### Beyond the strait of primary and specialized care facility sustainability: the harmonization of bottom-up and top-down processes

There is a trend toward the loss of adherence to the Mediterranean diet in the last two decades, in Italy, even not associated with BMI or physical activity change. Adherence to the Mediterranean diet is significantly yearly decreasing in Italy in healthy subjects [11,34]. Dietary, psychological and physical exercise counseling can affect adherence to healthy guidelines and outcome in obesity, e.g. relationship of these factors was studied. Behavioral counseling focused on physical exercise and self-efficacy enhances dietary adherence and weight loss in obesity [35].

**Figure 6:**
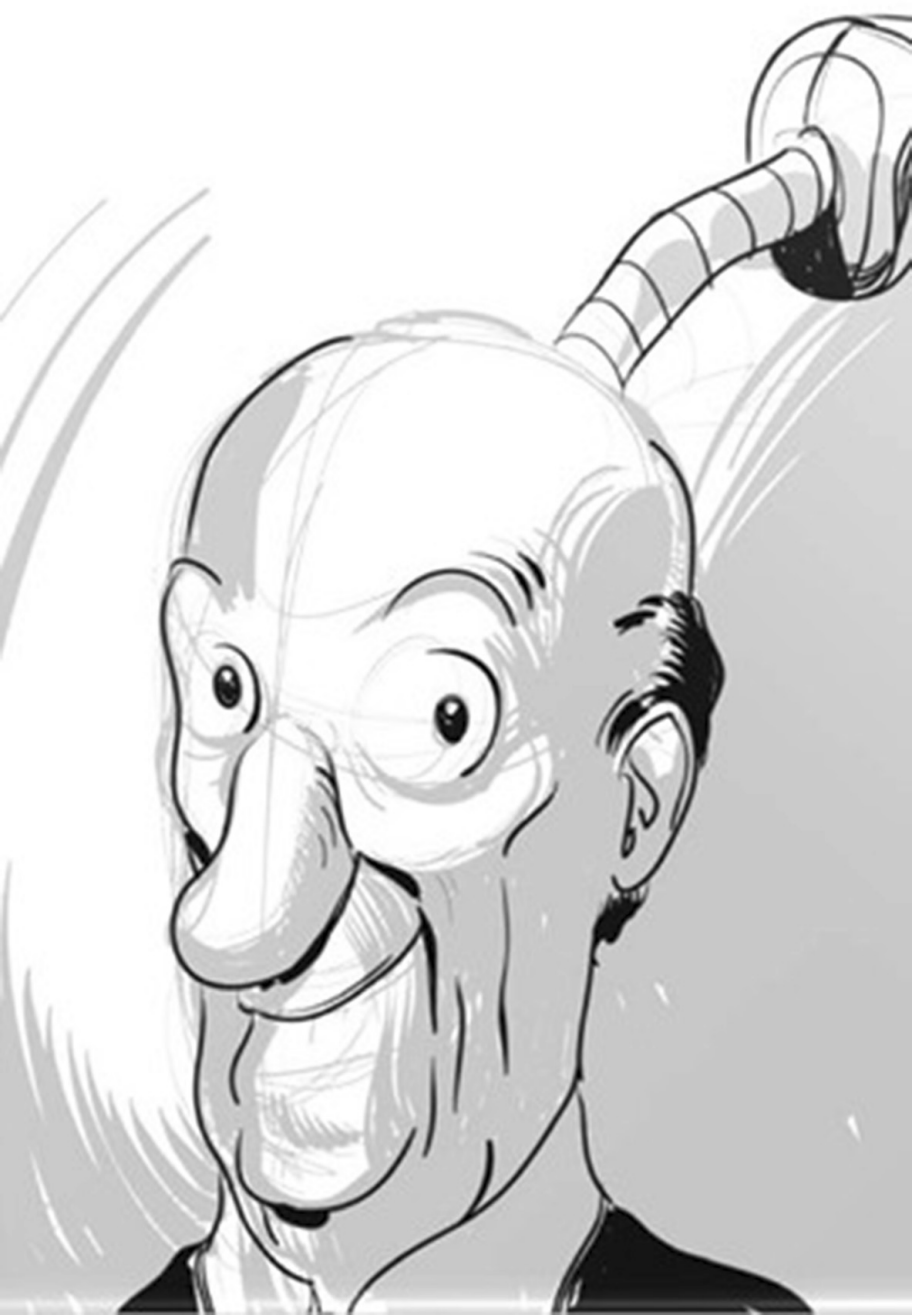
**The strategy for teaching and training predictive, preventive and personalized medicine does not mean that the mind of health professionals can be filled as a gasoline tank.** The intention to teach and the intention to train are the bases for achieving as many as possible affordable task forces, able to afford firmly and adequately, without worry and excessive involvement, complex conditions.

**Figure 7:**
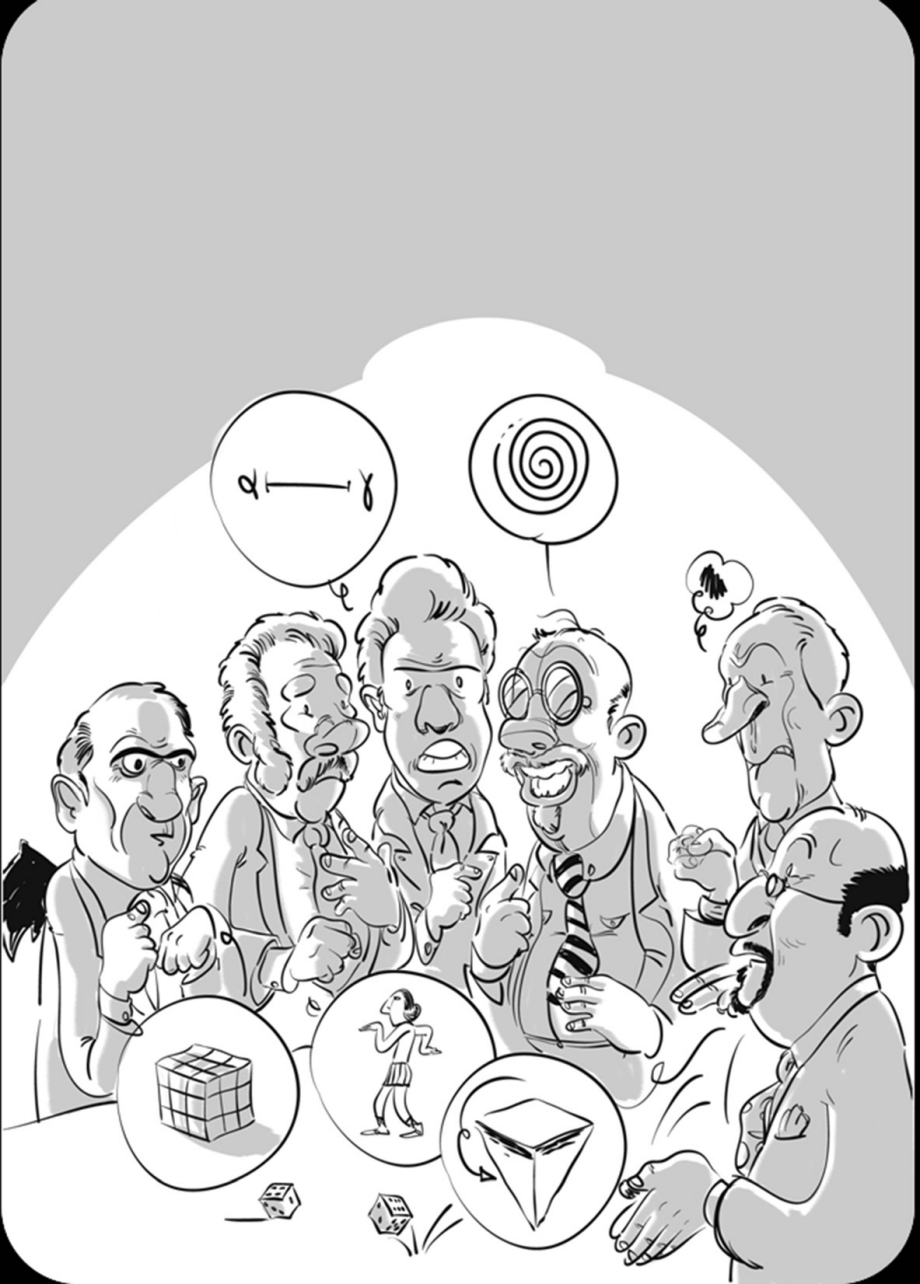
**Exploring the outpatient clinic added value, beyond the strait of primary and specialized care facility sustainability of general DTUs, is not only support for cancer survivors.** The consultations of many specialists, if not appropriately addressed and adequately driven, can lead to uncertainties of decisions and be more time-wasting than helpful.

Nutritional guidelines in arterial hypertension are presently not sufficiently followed in Italy as elsewhere: we investigated if dietary adherence of patients can be a consequence of the different health professionals involved. The need of an expert professional counseling on arterial hypertension was found necessary due to the co-existence of obesity and of the low adherence to suggested dietary guidelines, without space for non-professional clinical interventions: these are time- and money-wasting and their effects are often unfavorable. We found that beneficial changes can be achieved using the Mediterranean diet as a nutritional and friendly paradigm also in arterial hypertension [36]. Along this line, we collaborated within an intervention of the Italian Food and Nutrition Public Provincial Service, The Spontaneous Plants' Recipes Project, which was aimed to enhance Mediterranean diet profile change by a sustainable strategy for obesity prevention in childhood, contributing also to biodiversity maintenance [37]. The intervention was successfully performed with children by the collaboration of teachers, families and health professionals and was the natural complement of a precedent intervention, ‘The Grandmothers' Recipes Project’ , which was an integrated comprehensive intervention aimed at counteracting the obesity epidemics in childhood by promoting knowledge and skills in the Mediterranean diet [38]. Also, the relationship of an accurately designed intervention with different diseases (liver, neurological, cardiovascular) is a challenge that can be appropriately reported for dissemination [39-43]. In the domain of health care, knowledge, theories and practice are far from being established and much is still to be known about how something might work better and new ideas for improvement are needed. By research, the findings can be recorded numerically and then statistically analyzed in order to determine whether the results are significant (i.e. the extent to which it can be claimed with a specified degree of certainty that they are not just due to chance). Moreover, novel, controversial, sensitive or taboo issues need to be studied, also to give a voice to vulnerable or minority groups.This is the reason for which we are actively promoting interdisciplinary knowledge, also involving the cultural stakeholders that can be committed to improve the health literacy of our populations. Misleading or actually fake messages and information are of great concern everywhere since their influence is detrimental not only to healthy and sick people but, equally, to all health professionals. The strategy for teaching and training predictive, preventive and personalized medicine does not mean that the mind of health professionals can be filled as a gasoline tank. The intention to teach and the intention to train are the bases for achieving as many as possible affordable task forces, able to afford firmly and adequately, without worry and excessive involvement, complex conditions (Figure 6). Also, consultations of many specialists, if not appropriately addressed and adequately driven, can lead to uncertainties of decisions and be more time-wasting than helpful (Figure 7).

### What, who, when, where, why and how: the process of an affordable PPPM with a focus on youngsters

**Figure 8:**
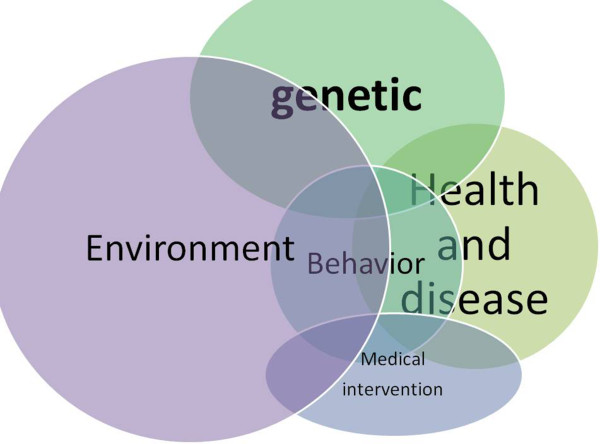
**Health and disease main determinants are genetic, environmental and behavioral.** The comprehensive concept of environment which includes geography, climate, anthropic modification, urban, rural and occupational subsets should be considered, along with the societal issues. Relationship with individual genetics and behavior is modulated by interactions with environment and its political and economical effectors. Looking for what, who, when, where, why and how is the frame of any investigation.

**Figure 9:**
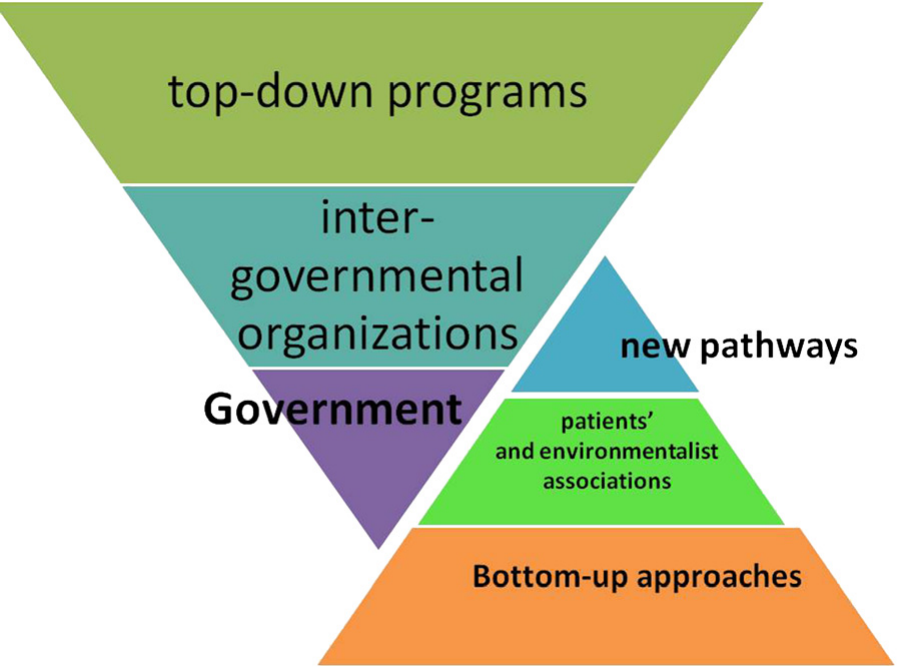
**The harmonization of bottom-up and top-down processes is the cornerstone for the enhancement of research within a health institution.** Both top-down and bottom-up approaches exist in public health. The most common are the top-down programs, often run by governments or, at a lower extent, by large inter-governmental organizations; bottom-up programs include many small non-governmental organizations and patients' and environmentalist associations. Health professional organizations are set up to improve local or general access to health care and the quality and efficacy of clinical procedures and even to open new pathways for basic and translational research.

Health and disease main determinants are genetic, environmental and behavioral (Figure 8). Looking for what, who, when, where, why and how, as in any investigation, for the side of environment geography, climate, anthropic modification, urban, rural and occupational subsets should be considered, along with the societal issues: relationship with individual genetics and behavior, variously modulated and interacting with environment and its political and economical implication, deserves great attention and is the actual objective of research with a perspective of predictive, preventive and personalized medicine.The harmonization of bottom-up and top-down processes is the cornerstone for the enhancement of research within a health institution (Figure 9). These two major approaches to many fields of design and planning of a research are also two different strategies of information achievement and processing with the need of harmonization of implementation and subsequent knowledge ordering. Actually, we are dealing with different styles of thinking, teaching and working in research.

By a bottom-up approach, investigation works from a large number of people working together, with direct and front-line experience and with individual independent and creative thinking; this approach implies a realistic and timely sequence of steps able to reach a decision to arise from a joint involvement and from the choice of a shared idea and objective, which need to be investigated and developed. A bottom-up approach allows for more innovative experimentation and for a better feeling also for what is perceived to be needed at the bottom. The assessment and prioritization of the bottom-up proposal must lead to a coherent top-down approach in which decision-makers disseminate and implement interventions, also with research goals, under their authority toward lower levels in the hierarchy. Both top-down and bottom-up approaches exist in public health. The most common are the top-down programs, often run by governments or, at a lower extent, by large inter-governmental organizations; bottom-up programs include many small non-governmental organizations and patients' and environmentalist associations. Health professional organizations are set up to improve local or general access to health care and the quality and efficacy of clinical procedures and even to open new pathways for basic and translational research. Institutional and private research funding actions can privilege the one or the other approach.

**Figure 10:**
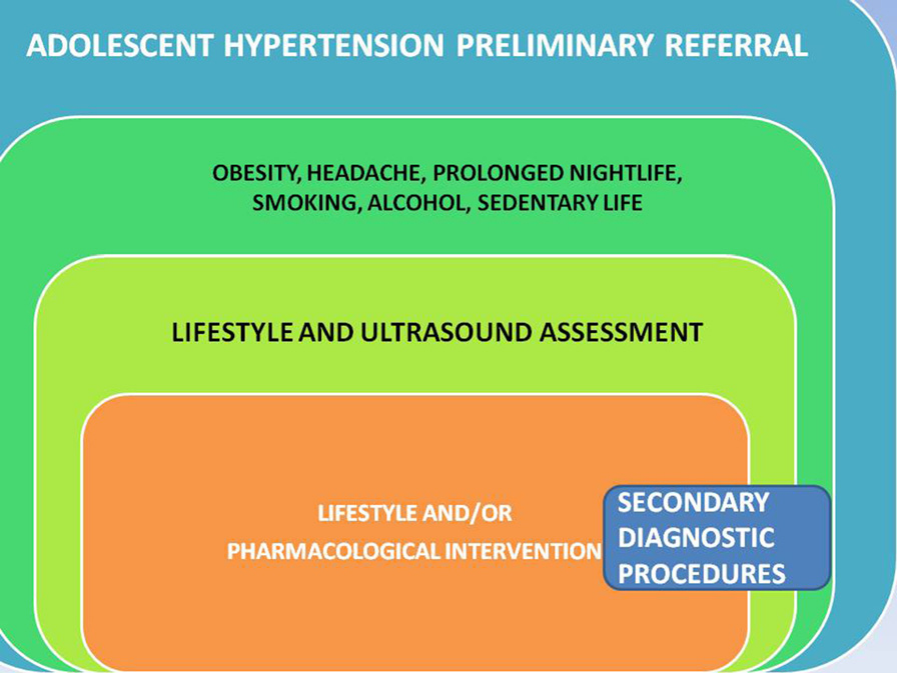
Work-up for adolescent hypertension assessment and intervention.

The model of PPPM of adolescent hypertension, which we developed since 1976 and is still active, was displayed in our course as an effective and clinically rewarding approach which is still a current health and societal appeal included in most research calls. This is a paradigm of comprehensive PPPM aimed at the management of a recognized, but actually scarcely implemented, societal and clinical topic. Environmental factors, even in the urban context, are important and can be studied with a close link with severe disease and risk factors, including excessive leisure nightlife—*mala****-****movida*—and excessive exposure to leisure noise; the link between sleep deprivation, noise exposure and the more commonly considered unhealthy habits is one of the possible new frontiers of medical intervention in any age but mainly in youngsters [44,45]. The lasting approach of our research group with a focus on lifestyle intervention and assessment linked with an affordable strategy of early diagnosis is well itemized by the work-up for the assessment of juvenile-adolescent hypertension (Figure 10). Following our earlier reports [46-48] in 1976–1979, by which the epidemiological features of adolescent hypertension were studied in a group of 1,060 youngsters aged 14–19 years, we performed a subsequent 2-year follow-up with lifestyle intervention addressed to alcohol and smoking withdrawal and to healthy nutrition and physical exercise counseling. Results were favorable, in the short-medium term, with the achievement of significant weight normalization (from obesity and underweight toward normal body weight) and of the decrease of blood pressure of subjects which were within the arterial hypertension range. Thereafter, since 30 years, the outpatient clinic served also as the reference point of primary care doctors for patients with juvenile hypertension. Ultrasound assessment of renal function circulation and malformation, the US detection of aortic coarctation and the US investigation of nodules possibly responsible for endocrine hypertension (thyroid, ovary and adrenal gland disease, pheochromocytoma, paragangliomas), along with the more usual echocardiography for left ventricular hypertrophy, were the key components of our approach and training, preliminary to other investigations and to management [49-51]. Patients' needs, innovative medical sciences, optimal health and disease management, expert recommendations for the relevant medical fields and optimal solutions which have a potential to advance health care services if the long-term strategies were to be effectively implemented [52,53] must begin from intervention addressed to youngsters using clinical strategies based on the best evidence available and on actual comprehensive expertise, as proposed here. This approach is still a matter of discussion, as a new one, worldwide [54]. We are actually putting into practice this clinical strategy since four decades, and the rationale was based on what, who, when, where, why and how:

•What: arterial hypertension, associated or not with obesity, as a factor of cardiovascular disease, disability and death.

•Who: persons at risk of early development of unhealthy lifestyle and disease.

•When: adolescents in Italy are conventionally 14–18 years aged, i.e. still in the pediatric range for the US criteria.

•Where: in different core phases of their life, i.e. at home, school, work, sport and, importantly, leisure time.

•Why: assessment of arterial hypertension and obesity, as clinical biomarkers, along the occurrence of symptoms and unhealthy behavior, such as late bedtime and prolonged nightlife, provides information and suggestion for effective intervention.

**Figure 11:**
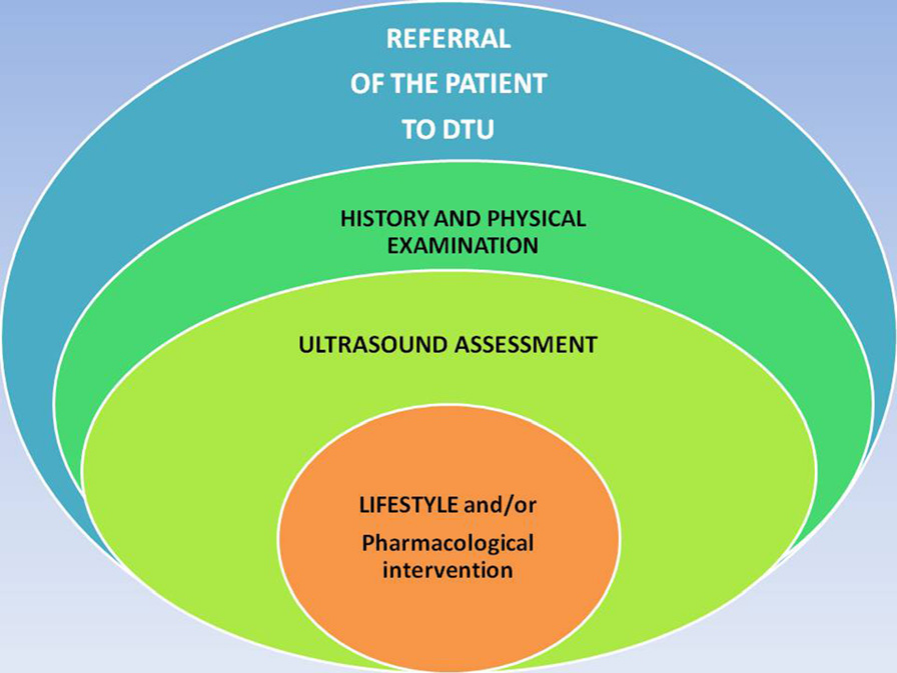
Referral to the DTU.

•How: after the preliminary assessment and dissemination of information [46-48], primary care doctors, teachers and the general population acquire the awareness of the dangers of obesity and arterial hypertension in the youngsters; understand some of the related symptoms, such as day sleepiness and, mostly, headache; and refer those likely at risk to single specialists or to a centre with facilities for a comprehensive approach.This sequence of 5Ws and How is the daily object of reappraisal by our group, considering the actual yield of information provided by the most comprehensive tools: basic laboratory examination; EKG; echocardiography and thyroid, vascular and abdominal-renal-thoracic ultrasound, the latter being delivered jointly with physical examination by an expert MD expressly committed to this diagnostic-therapeutic intervention. A cost-benefit analysis is performed, with a favorable outcome. This work is allowed despite that the current rules and managed care theory and practice favor more expensive approaches which rely on multidisciplinary specialist consultations and on stepwise requests and feedbacks, with a consequent wasting of time and human and financial resources (Figure 11).

## Conclusion

**Figure 12:**
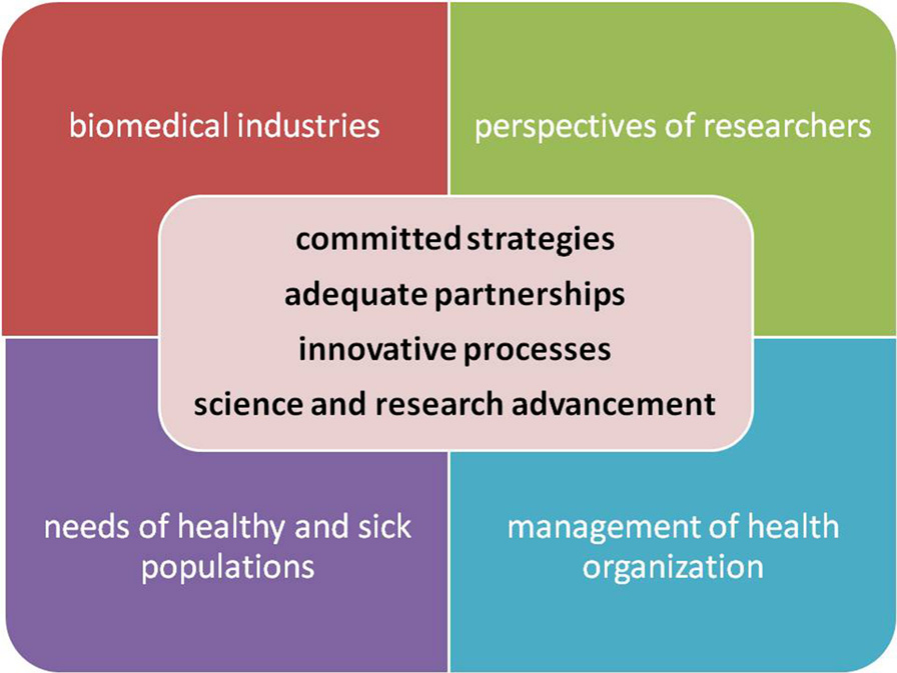
**The task of a scientific community, committed to the promotion of predictive, preventive and personalized medicine, is to facilitate the road of bottom-up approaches, stemming not only from the actual needs of healthy and sick populations but also from the perspectives of researchers able to explore novelty and the unknown, before and behind translational research.** The management of health organization and biomedical industries should interact in order to build up strongly committed strategies and adequate partnerships for achieving science and research advancement, also establishing innovative processes.

The task of a scientific community, committed to the promotion of predictive, preventive and personalized medicine, is to facilitate the road of bottom-up approaches, stemming not only from the actual needs of healthy and sick populations but also from the perspectives of researchers able to explore novelty and the unknown, before and behind translational research (Figure 12). This task must be shared with the appropriate stakeholders and, mainly, with the management of health organization and biomedical industries in order to build up strongly committed strategies and adequate partnerships. The current top-bottom approach not only has the inherent risk of desertification of the potentiality of science and research advancement in medicine but also is an obstacle to the establishment of innovative processes. Health professionals should and can be skilled in sustainable non-invasive diagnostic procedures, in non-pharmacological intervention, in translational research (from epidemiology to personalized therapy) and in timely dissemination of the information.

We need to guide and prioritize this process, shifting emphasis toward the principles of evidence-based medicine, acknowledging that evidence may still be misinterpreted or distorted by recalcitrant proponents of entrenched practices and other biases [33].

The quality of a good research can be undermined by several obstacles which hamper affirming new results and conclusions; this will slow down innovation and development, and when it has happened, and happens, misleading and badly supported concepts and methods enter in use, warranted and recommended by official institutions and scientific associations. The rules of the evidence-based medicine can be distorted by artifactual premises and goals. When concepts and methods are established by this means, and supported by plethora of studies and articles, even redundant but assumed as confirmatory, the road for the establishment of realistic approaches and reliable concepts is tortuous. Medical reversal happens when new trials better powered, designed or controlled than predecessors contradict the current standard of care. These reversals are instructive in that many of the therapies overturned were widely adopted and based on either sound physiologic reasoning or observational trials [33]. The need of well-planned projects to contradict wrong concepts is obvious, but the strategy for making it realistic is not easy. The first steps could be to polemicize by timely and well-argumented letters and commentary in the same journals which continue to publish highly questionable reports of research or even only case reports. This is a point more important than commonly appreciated, since by this strategy the editorial boards and, much more, the editors in chief are warned and some of the readers will take part, with their specific ideas and experience, in this dialectic intervention, also with further and better addressed researches.

Innovative research idea incubators are sustainable environments that can be enhanced in many and even small institutions. Medical research incubators differ from research and technology parks in their dedication to enhancing independent thinking against several current problems and biases, particularly in clinical medicine, and provide support to early-stage small-scale projects. The training is the systematic assessment of research project-based scientific articles in the specific fields of research and practice, to find defects, contradictions or, not infrequently, clues of fake reports and to react, if needed, addressing letters or commentary to the editors. Editors, as a rule, appreciate, if not explicitly solicit, this type of volunteer contribution, which is less easy to provide addressing to open-access pay-for-publish journals [55]. ‘The truth isn't always beauty, but the hunger for it is’ (Nadine Gordimer). It is still true that it is useless to fight a battle if you do not gain anything by winning: the gain can be not immediate or direct, but simply a tool for warning editors and readers about the possibility that some report could be at least questionable: Perplexity is the beginning of knowledge. In research, ‘there are two possible outcomes: if the result confirms the hypothesis, then you've made a measurement. If the result is contrary to the hypothesis, then you've made a discovery’ (Enrico Fermi). The third one is ‘no measure, no discovery’. Also, a null result in clinical research, as in many studies should happen, can be a useful contribution for better managing the overflow of different conclusions and information, with futile or misleading recommendations. And a timely remark, everywhere, but better on the same journals that published articles with which there is no concept or methodological agreement, is the most affordable and effective tool. Along this line, ‘we are not sadistic scientists who hurry to hunt down errors instead of establishing the truth. In science, we must be interested in things, not in persons, being less curious about people and more curious about ideas.’ (Marie Curie). A research school is actually very similar to a peace strategy school.

Research counseling is today more addressed to the way of presenting a mature proposal and to find prominent/well-acknowledged partners but less to enhancing truly innovative ideas which are usually considered as being too risky. Academic research has too often the features of a tool devised more for enhancing niche knowledge and career progress, or to use funding for current activities, than as an actual resource for promoting quality in health care and advancement in knowledge.

We must acknowledge that there is the need to reconsider the policy of granting and the rules of funding by better matched expertise in the task of evaluation of proposals. Commitment should be focused on effective health promotion, predictive/early diagnostics of the disease and treatments tailored to the person. In particular, a special attention should be paid to the principles of reaching the agreement in the evaluation panel and/or mechanisms which allow for reconsidering the initial scores in problematic panels. These rules, in several European Member States and even in EU are frequently not sufficiently clearly defined. Consequently, current granting policy is not effective enough to support consolidated efforts for enhancing skills and knowledge in the innovative medical fields such as predictive, preventive, personalized and participatory medicine which, nonetheless, is considered as the medicine of the future. The contribution of the best committed experts and matched expertise in the evaluation panel is strongly required.

## Expert recommendations

PPPM experience and proposed models need to be monitored and assessed not only on the basis of financial sustainability and patient's satisfaction, as currently is done by public and private institutions, but also by carefully designed research projects. These must take adequately into account different perspectives of impact and actual outcomes, with a wise articulation of bottom-up and top-down approaches.

Guidelines of the EU health research calls addressed to personalized medicine, in its wider meaning and applications, are able to add value to any activity, by the requirements of accurate planning, transparency of assessment and unbiased reports, dissemination and exploitation. The actual pre-requisite of personalized medicine is the coherent and articulated promotion of the professional quality of staff.

Any project dealing with health and disease should include an effective training of professionals toward these PPPM directives and planned articulated collaborations with academy, health institutions, cities, non-profit organizations and SMEs. With this professional and scientific background, and a greater awareness and endorsement also of patients' associations, it will be possible to rely on more realistic goals for better supporting healthy and sick citizens, such as Lasting Cancer Survivors.

## Competing interests

The author declares that he has no competing interests.
